# A MYB transcription factor, *BnMYB2*, cloned from ramie (*Boehmeria nivea*) is involved in cadmium tolerance and accumulation

**DOI:** 10.1371/journal.pone.0233375

**Published:** 2020-05-18

**Authors:** Shoujing Zhu, Wenjuan Shi, Yucheng Jie, Qingming Zhou, Chenbo Song

**Affiliations:** 1 Institute of Ramie, Hunan Agricultural University, Changsha, Hunan, China; 2 College of Life Sciences, Resources and Environment, Yichun University, Yichun, Jiangxi, China; National Taiwan University, TAIWAN

## Abstract

MYB-related transcription factors play important roles in plant development and response to various environmental stresses. In the present study, a novel MYB gene, designated as *BnMYB2* (GenBank accession number: MF741319.1), was isolated from *Boehmeria nivea* using rapid amplification of cDNA ends (RACE) and RT-PCR on a sequence fragment from a ramie transcriptome. *BnMYB2* has a 945 bp open reading frame encoding a 314 amino acid protein that contains a DNA-binding domain and shares high sequence identity with MYB proteins from other plant species. The *BnMYB2* promoter contains several putative cis-acting elements involved in stress or phytohormone responses. A translational fusion of *BnMYB2* with enhanced green fluorescent protein (eGFP) showed nuclear and cytosolic subcellular localization. Real-time PCR results indicated that *BnMYB2* expression was induced by Cadmium (Cd) stress. Overexpression of *BnMYB2* in *Arabidopsis thaliana* resulted in a significant increase of Cd tolerance and accumulation. Thus, *BnMYB2* positively regulated Cd tolerance and accumulation in *Arabidopsis*, and could be used to enhance the efficiency of Cd removal with plants.

## Introduction

With the rapid development of industry and mining, excessive emission of industrial waste water, gas and residue containing heavy metals (Cu, Pb, Cd, As, Zn and so on) has resulted in serious pollution of farmland soil [1, 2]. Cadmium (Cd) is a highly toxic metal element, with high mobility, accumulation and non-degradability, that is considered non-essential for living organisms. Once exogenous Cd gets into the soil, it progressively accumulates and causes long-term harm to farmland and crops [3, 4]. More importantly, Cd may jeopardize food security and human health through food chain bioaccumulation [5]. Research on remediation techniques for Cd polluted farmlands has increased since Cd pollution was reported to have caused "Itai-itai" disease in Japan in 1961 [6].

Phytoextraction has been promoted as an environmentally-friendly, low-input alternative for remediation of soil contaminated with heavy metals [7–10]. Recently, plant species used for phytoremediation have been extended from hyperacuumulators to non-hyperaccumulators such as *Brassica napus* [11], *Solanum nigrum* [12], *Boehmeria nivea* [13] and *Helianthus annuus* [14], which have significantly higher growth rates and biomass than hyperaccumulators. However, the use of these species is often limited by low levels of heavy metal accumulation related to their lower tolerance to heavy metals. Use of genetic engineering technology to increase heavy metal tolerance, uptake and accumulation in plants has expanded new methods in phytoremediation.

Among several mechanisms of toxicity, Cd is known to markedly increase oxygen free radicals and reactive oxygen species (ROS) in plants; to induce the oxidation of protein, DNA, lipid and carbohydrate; and to inhibit many biological and biochemical processes in organelles through its high affinity for sulfhydryl groups of proteins [15–19]. Transcriptional regulation contributes to plant response to environment stresses [20]. Transcription factors (TFs) regulate diverse physiological and biochemical responses to different abiotic stresses by activating a variety of stress-related genes [21].

The MYB (v-myb myeloblastosis viral oncogene homolog (avian)) gene family exists widely in many eukaryotes and is the largest transcription factor family in plants. All MYB proteins contain MYB domains that are highly conserved in animals, plants and yeast [22]. The MYB domain is comprised of 51–52 amino acid residues. In accordance with the number of MYB domain repeats, plant MYB proteins have been classified into 4 subfamilies: 1R-MYB/MYB-related, R2R3-MYB, R1R2R3-MYB and 4R-MYB [23, 24]. MYB genes play important roles in abiotic stress response in plants [25, 26]. Up-regulation of *OsMYB48-1*, an *Oryza sativa* MYB transcription factor, promotes drought tolerance in rice by influencing ABA synthesis [27]. Transgenic *Arabidopsis* (*Arabidopsis thaliana*) overexpressing *OsMYB3R-2* exhibit enhanced cold stress tolerance [28]. *AtMYB15* overexpression in transgenic *Arabidopsis*, which negatively regulated the expression of *CBF* genes, improved tolerance to cold [29] and drought stress [30]. Excessive copper ions (Cu^2+^) in rice significantly influenced the expression of several kinds of transcription factors, including MYB [31]. The R2R3-MYB transcription factor *OsARM1* was implicated in arsenic (As) stress response in rice [32]. About 20 percent of all MYB family genes in *Arabidopsis thaliana* are differentially expressed in response to Cd stress [33]. Overexpression of the Cd-induced *MYB49* gene in *Arabidopsis* resulted in a significant improvement in Cd accumulation, whereas *MYB49* knockout plants and plants expressing chimeric repressors of *MYB49*: ERF exhibited reduced Cd accumulation [34]. In ramie, the transcript levels of fourteen MYB transcription factor genes were found to be affected by Cd stress. The expression of seven MYB genes were up-regulated and seven genes were down-regulated, respectively [35].

Ramie (*Boehmeria nivea* L.), also known as “China grass”, is a perennial herb from the Urticaceae family with a large shoot biomass, fast growth rate and strong root system [36]. Ramie is one of the most important natural fiber crops and can usually be harvested three times per year. It is widely cultivated in China, India, and other Southeast Asian and Pacific Rim countries. In southern China, ramie has been cultivated for at least five thousand years, yielding fiber production that is second only to cotton. Previous studies have indicated that ramie has strong tolerance to and ability to accumulate certain heavy metals, such as Cd, Pb and As, from soil [36–38]. More importantly, ramie can accumulate a relatively large amount of heavy metals in its aboveground parts. The mean concentration (mg·kg^-1^) of Cd, Pb and As in leaves is 2.7, 27.2 and 55.6, respectively, under field conditions [39]. In addition, it is a good candidate for phytoextraction of heavy metal polluted soils because its fiber is not in the food chain and is easy to harvest. In our previous studies on the transcriptome profiling of cadmium response genes in ramie, unigene35788 was found to be significantly up-regulated in cadmium treated groups, which was annotated as MYB transcription factors using NR and KEGG database [40]. MYB transcription factors play critical roles in plant resistance to environment stress. However, scant research on the function of MYB genes in ramie has been reported. This study focused on the identification of a putative MYB transcription factor (*BnMYB2*) from ramie and analysis of its promoter, expression profile, subcellular localization and transgenic overexpression in order to investigate the function of this gene in Cd stress response in ramie.

## Materials and methods

### Plant materials and stress treatments

*Boehmeria nivea* cultivar Zhongzhu-1 used as plant material in this research was grown in the experimental farm at Yichun University, Yichun, China. The 20-day-old ramie seedlings were grown in perlite pots and irrigated with half strength Hoagland solution. For tissue specific expression analysis, roots, stems and leaves from 20-day-old ramie seedlings were collected separately and stored at -80°C until used. To assess the effects of various Cd exposure times, seedlings were irrigated with Cd solution (100 μM) for 0, 3, 6, 12, 24 or 48 hours, and then leaves were sampled and stored at -80°C until RNA extraction. For treatments with varying Cd concentration, seedlings were irrigated with solutions with different Cd concentrations (0, 10, 30, 50,100 and 150 μM) for 24 hours, and then the leaves were sampled, frozen in liquid nitrogen and stored in -80°C until RNA extraction.

### Isolation of full length *BnMYB2* gene

From transcriptome screening, the unigene35788 was identified with BLAST (https://blast.ncbi.nlm.nih.gov/Blast.cgi) against National Center for Biotechnology Information (NCBI) nucleotide database based on its homology with known MYB transcription factors. Sequence analysis revealed that both the 3’ and 5’ ends of unigene35788 were incomplete. Therefore, full-length *BnMYB2* cDNA was obtained by performing 3’ RACE using primer MYB2-3FO/3FI and 5’ RACE using primer MYB2-5RO/5RI (S1 Table) with UAP primer and instructions provided by the SMARTer RACE 5’/3’ Kit (Clontech, USA). PCR products were run on a 1.5% agarose gel and then purified with the MiniBEST Agarose Gel DNA Extraction Kit (TaKaRa, China). Next, purified PCR product was inserted into pMD19-T plasmid (TaKaRa, China) and sequenced at the Shanghai Shenggong Company (Shanghai, China). In order to amplify full-length *BnMYB2* cDNA, a pair of specific primers MYB2-F/MYB2-R (S1 Table) was designed on the basis of full-length sequence obtained from an alignment of the partial sequence, 3’RACE product and 5’RACE product. The PCR amplification procedure was 5 min at 94°C, 32 cycles (30 s at 94°C, 30 s at 52°C and 90 s at 72°C) and final extension for 7 min at 72°C. The PCR product was run on a 1.5% agarose gel, purified with a MiniBEST Agarose Gel DNA Extraction Kit (TaKaRa, China) and sequenced at the Shanghai Shenggong Company (Shanghai, China). The *BnMYB2* nucleotide sequence was submitted to GenBank (accession number: MF741319.1).

### Sequence analysis

ORF Finder in the NCBI website (https://www.ncbi.nlm.nih.gov/orffinder/) was used to search the *BnMYB2* open reading frame. The basic physical and chemical properties of the deduced amino acid were analyzed with ProtParam tool in the ExPASy website (https://web.expasy.org/protparam). Multiple sequence alignment was performed with DNAMAN software (version 6.0) set to default parameters. The conserved domains of the BnMYB2 protein were predicted using the Conserved Domain Search (https://www.ncbi.nlm.nih.gov/Structure/cdd/wrpsb.cgi). The phylogenetic tree analysis was performed with MEGA software (version 5.0) using the neighbor-joining (NJ) method (1000 replication of bootstrap) according to Kimura 2-parameter distance with BnMYB2 and other species provided in Genbank of NCBI. Protein subcellular location was predicted using WoLF PSORT (https://www.genscript.com/wolf-psort.html).

### Isolation of the *BnMYB2* promoter

Genomic DNA was extracted from the fresh ramie leaves. Specific primers MYB2-PF/MYB2-PR (S1 Table) were designed to clone the 1947 bp upstream of the *BnMYB2* initiation codon. PCR cycling conditions were 5 min at 94°C, 32 cycles (30 s at 94°C, 30 s at 59°C and 2 min at 72°C) and final extension for 7 min at 72°C. PCR product was run on a 1.5% agarose gel, purified with a MiniBEST Agarose Gel DNA Extraction Kit (TaKaRa, China) and sequenced at the Shanghai Shenggong Company (Shanghai, China). Putative cis-acting elements in the *BnMYB2* promoter were identified using the PlantCARE database (http://bioinformatics.psb.ugent.be/webtools/plantcare/html).

### Subcellular localization of BnMYB2 protein

The complete coding sequence of *BnMYB2* was amplified using MYB2-XbaI-F and MYB2-BamHI-R primers with a *Xba* I site and a *BamH* I site (S1 Table). Then, the PCR products were digested with *Xba* I/*BamH* I and inserted into pAN580 vector containing a 35S promoter-driven GFP reporter gene. The 35S::OsGhd7-CFP plasmid was used to produce the nuclear marker while the empty vector 35S::GFP was used as control [41]. The constructs was sequenced and transformed into *Arabidopsis* protoplasts. Protoplast isolation and transfection procedures were performed according to the method described by Yoo et al. [42]. Briefly, mesophyll protoplasts were isolated from rosette leaves collected from 3 to 4-weeks-old Col wild type *Arabidopsis thaliana* plants. Then, the nuclear marker construct 35S::OsGhd7-CFP was co-transformed with 35S::BnMYB2-GFP construct or 35S::GFP construct into *Arabidopsis* protoplasts. Finally, the GFP and CFP fluorescences were observed using Olympus FV1200 confocal microscopy imaging system after the protoplasts were incubated at room temperature for 20–22 h under darkness.

### Tissue-specific and Cd-induced *BnMYB2* expression

To analyze the tissue-specific expression pattern of *BnMYB2*, total RNA was extracted from ramie roots, stems and leaves using TRIzol reagent. The PrimeScript RT reagent kit with gDNA Eraser (TaKaRa, China) was used for cDNA synthesis by following the manufacturer’s instructions. For gene expression analysis under Cd treatment, total RNA was extracted from leaves harvested from Cd-treated ramie seedlings. Then total RNA was reverse-transcribed into first strand cDNA in accordance with the user’s manual. Quantitative real time PCR (qRT-PCR) was performed in triplicate using TB Green Premix Ex Taq II kit (TaKaRa, China) in the StepOnePlus Real-Time PCR System (Applied biosystems) by following the manufacturer’s instructions. The primers used were MYB2-qF/MYB2-qR and BnActin-F/BnActin-R (S1 Table). The relative quantification method (ΔΔ^-CT^) was used to quantify the relative expression normalized to the reference gene (β-actin) [43]. The qRT-PCR assays were conducted with three biological replicates and three independent technical replicates for each sample.

### Construction of plant expression vector and *Arabidopsis thaliana* transformation

The *BnMYB2* ORF was cloned with the specific primers MYB2-BamHI-F and MYB2-SacI-R (S1 Table) and subcloned into pBI121, a plant expression vector carrying the CaMV35S promoter, to construct the fusion plasmid pBI121-BnMYB2. Then, recombinant plasmid was transformed into *Arabidopsis thaliana* cultivar Columbia-0 using *Agrobacterium tumefaciens* strain EHA105 in the floral dipping method. Transformants (T1 generation) were selected by planting seeds on MS plates containing 30 mg/L kanamycin. Positive transformants were confirmed with genomic PCR using the MYB2-35SF/MYB2-SPR primers (S1 Table). For this, genomic DNA was isolated from independent *BnMYB2* overexpression and wild-type plants. The PCR cycling conditions were 5 min at 94°C, 32 cycles (30 s at 94°C, 30 s at 64°C and 30 s at 72°C) and final extension for 7 min at 72°C. To further confirm positive transformants, RNA extracted from leaves of transformants (T2 generation) was reverse-transcribed into cDNA, and then RT-PCR was performed as described above.

### Cd stress assay

#### Phenotypic observation

To test the effect of Cd on the growth of transgenic *Arabidopsis* seedlings, seeds of transgenic plants (T3 generation) and wild-type plants were sterilized with 70 percent ethanol and sodium hypochlorite, plated on MS (Murashige and Skoog) solid medium containing Cd (0, 100 or 150 μM) and grown for 14 days. Phenotypes were photographed, and then the fresh weight and root length were measured. Wild-type *Arabidopsis* served as a control. There were three experimental replicates.

#### Detection of Cd content in plant

Wild-type and transgenic *Arabidopsis* were planted in plastics pots with unpolluted soil and irrigated with half-strength Hoagland nutrient solution for 30 days. Then, CdCl_2_ salt was added to the half-strength Hoagland nutrient solution to the final concentration of 0 and 50 μM, and the plants were watered with the Cd solution one time for dose-response experiment. After 7 days of cultivation, the plants were harvested, washed with tap water and rinsed with deionized water three times. Then, the roots and shoots were separated and dried at 75°C for 3 days. After that, Cd in the samples was measured by flame AAS with HNO_3_-HClO_4_ digestion. There were three experimental replicates.

### Statistical analysis

Statistical analysis was conducted using SPSS version 17.0 and Microsoft excel 2013 software. All data were expressed as the mean of three biological replicates ± standard deviation (SD). Comparisons between different groups were tested by one-way ANOVA, followed by Student’s t-test. A p-value less than 0.05 was considered to be significantly different.

## Results

### Isolation and characterization of *BnMYB2*

Cd is a major environmental pollutant that affects human health and plant growth. Ramie could tolerate and accumulate Cd when grown on polluted soils [38, 39, 44]. Searching for novel genes involved in Cd tolerance and accumulation has become a priority in ramie breeding [45]. In our previous studies on the transcriptome profiling of cadmium response genes in ramie, unigene35788 was found to be significantly up-regulated in cadmium treated groups, which was annotated as MYB transcription factors using NR and KEGG database [40]. In the present study, a 1 733 bp full-length cDNA for this gene (named as *BnMYB2*) was isolated using 3’-RACE, 5’-RACE and RT-PCR methods. Sequence analysis revealed that the cDNA contains a 945 bp open reading frame encoding a 314 amino acid protein (S1 Fig). The physicochemical properties of the predicted BnMYB2 protein were analyzed in ExPASy database. The results indicated that the BnMYB2 protein has a molecular mass of 33.91 kDa and an isoelectric point value of 8.87. The secondary structure of the BnMYB2 protein was predicted using NPS@ server. The result indicated that BnMYB2 protein consists of α-helices (21.66%), extended strands (5.73%) and random coils (72.61%).

**Fig 1:**
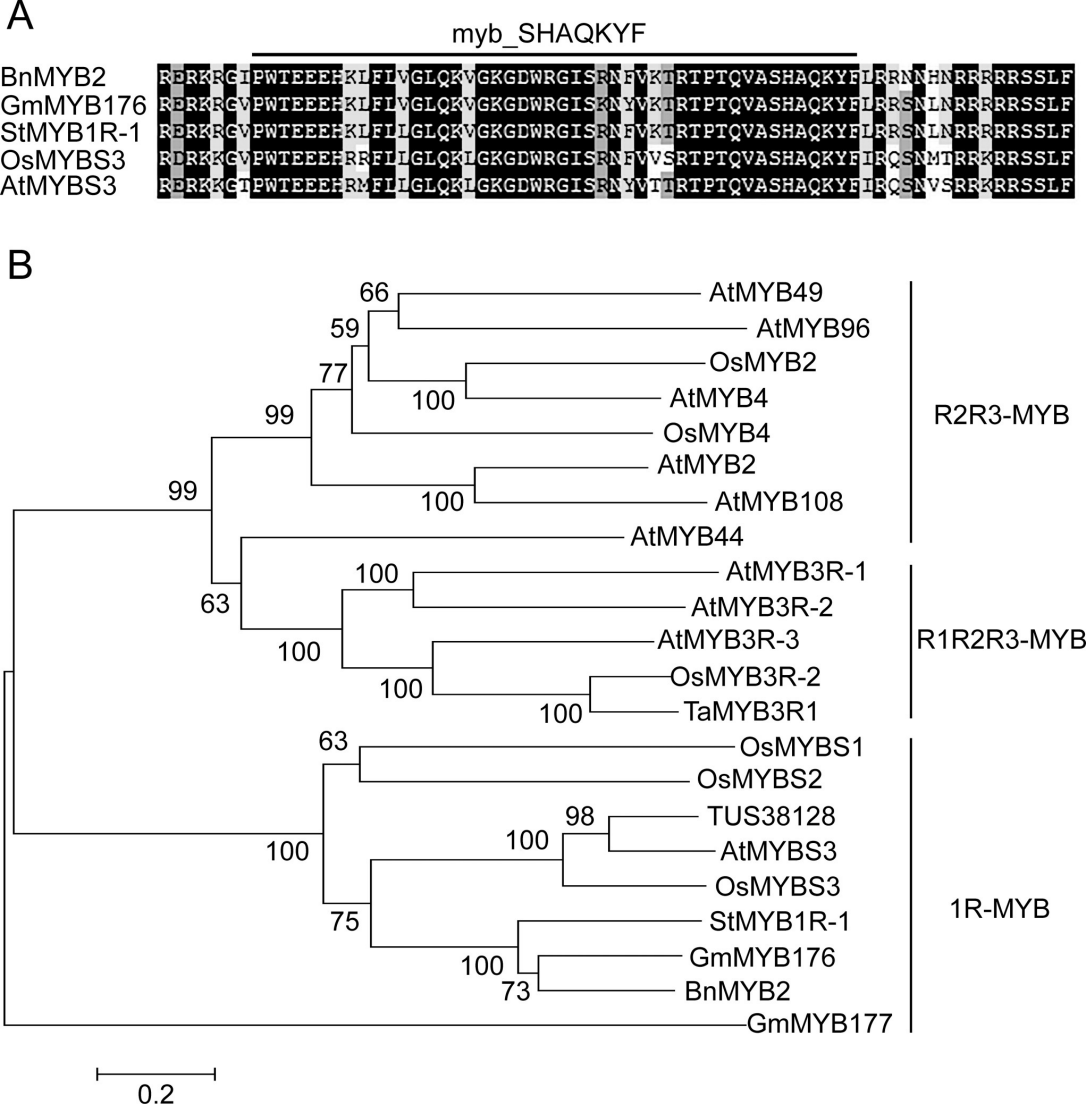
Multiple alignment and phylogenetic analysis of BnMYB2 and MYB proteins related to abiotic stress from other plants. **A**, The alignment of BnMYB2 and its ortholog proteins of different plants constructed using DNAMAN 8.0 software. Black shadow represents the conserved residues. The line indicates a highly conserved MYB DNA-binding domain (myb_SHAQKYF). **B**, Neighbor-joining phylogenetic tree of BnMYB2 from *Boehmeria nivea* and other MYBs constructed using MEGA 5.0 software. The scale bar represents 0.05 amino acid substitutions per site. The statistical reliability of individual nodes of the tree is assessed by bootstrap analyses with 1 000 replications. GenBank accession numbers of the proteins are as follows: AtMYB49 (At5g54230), AtMYB96 (At5g62470), AtMYB4 (At4g38620), AtMYB2 (At2g47190), AtMYB108 (At3g06490), AtMYB44 (At5g67300), AtMYB3R-1 (At4G32730), AtMYB3R-2 (At4g00540), AtMYB3R-3 (At3g09370) and AtMYBS3 (At5g47390) from *Arabidopsis thaliana*; OsMYB2 (BAA23338), OsMYB4 (BAA23340), OsMYB3R-2 (BAD81765.1), OsMYBS1 (AAN63152.1), OsMYBS2 (AAN63153.1) and OsMYBS3 (AAN63154.1) from *Oryza sativa*; TaMYB3R1 (ADO32617.1) from *Triticum aestivum*; TUS38128 (XP_004508939.1) from *Cicer arietinum*; StMYB1R-1 (ABB86258.1) from *Solanum tuberosum*; GmMYB176 (NP_001236048.2) and GmMYB177 (ABH02866.1) from *Glycine max*.

Conserved domain analysis showed that BnMYB2 contains a highly conserved MYB DNA-binding domain (myb_SHAQKYF) within residues 101–146, which indicated *BnMYB2* belongs to 1R-MYB subfamily. BLAST analysis showed that BnMYB2 matches with 1R-MYB proteins reported from *Solanum tuberosum* (NP_001236048.2), *Glycine max* (NP_001236048.2), *Oryza sativa* (AAN63154.1) and *Arabidopsis thaliana* (At5g47390) with approximately 65% identity at the amino acid level. Multiple alignment demonstrated that the MYB-DNA binding domain of 1R-MYBs from different plants are conserved (Fig 1A). Thus, this gene was designated as *BnMYB2* (GenBank accession number: MF741319.1), which was the second MYB gene identified in ramie.

BnMYB2 protein and twenty-one abiotic-stress-related MYB proteins from *Arabidopsis thaliana*, *Oryza sativa*, *Triticum aestivum*, *Cicer arietinum*, *Solanum tuberosum and Glycine max* downloaded from GenBank were used to construct a phylogenetic tree by the neighbor-joining (NJ) method with MEGA 5.0 software (Fig 1B). The results suggested that MYB proteins were divided into three groups (1R-MYB, R2R3MYB and R1R2R3-MYB). The BnMYB2 protein is more similar to TUS38128, AtMYBS3, OsMYBS3, StMYB1R-1 and GmMYB176 which belong to 1R-MYB transcription factor subfamily.

### Isolation and analysis of *BnMYB2* promoter sequences

A 1947 bp *BnMYB2* promoter was obtained from *Boehmeria nivea* by PCR amplification with a pair of specific primers (S2 Fig). The promoter sequence was analyzed using PlantCARE software (Table 1). The results indicated that the *BnMYB2* promoter contained the CAAT-box and TATA-box elements, which are typical core eukaryotic promoter regions. In the *BnMYB2* promoter sequence, two abscisic acid response elements (ABRE) were found at +1120 and +1767 bp, one anaerobic regulatory element (ARE) was found at -866 bp, one MeJA response element (CGTCA-motif) was found at +756 bp, one cis-acting element associated with circadian rhythm was found at +24 bp, one ethylene response element (ERE) was found at -957 bp, two gibberellin response elements (P-box) were found at +232 and -1732 bp, one stress response element (STRE) was found at -1822 bp and four wound response elements (WUN-motif) were found at +1162, +1395, +1227 and +1548 bp. The above analysis suggested that *BnMYB2* could respond to different plant hormones and multiple environmental stresses.

**Table 1:** Putative cis-acting regulatory elements identified in the *BnMYB2* promoter sequence using the PlantCARE database.

Cis element	Position	Sequence	Function of site
ABRE	+1120,+1767	ACGTG	cis-acting element involved in the abscisic acid responsiveness
ARE	-866	AAACCA	cis-acting regulatory element essential for the anaerobic induction
AT-rich element	+29	ATAGAAATCAA	binding site of AT-rich DNA binding protein (ATBP-1)
ATCT-motif	-867	AATCTAATCC	part of a conserved DNA module involved in light responsiveness
Box 4	+39, +760	ATTAAT	part of a conserved DNA module involved in light responsiveness
CAAT-box	+67,-271,+130,+202,+311,+318,+326,-489,-500,+884,-894,-908,-928,-950,+982,-1001,-1007,+1025,+1053,+1115,-1147,+1265,+1394,+1430,-1433,+1614,+1683	CAAT, CAAAT, CCAAT	common cis-acting element in promoter and enhancer regions
CGTCA-motif	+756	CGTCA	cis-acting regulatory element involved in the MeJA-responsiveness
circadian	+24	CAAAGATATC	circadian
ERE	-957	ATTTTAAA	ethylene-responsive element
G-Box	-1766,+1119	CACGTG, TACGTG	cis-acting regulatory element involved in light responsiveness
I-box	+1307	GATAAGGGT	part of a light responsive element
P-box	+232,-1732	CCTTTTG	gibberellin-responsive element
STRE	-1822	AGGGG	stress response elements
TATA-box	-45,+47,+103, +124, -125, +126, +172,+265, -302, +303,-445, +447, +464,-555,+556, 577, -609, +610,-739,-819, +822,-964,967,-1064, -1156,-1201,-1442, +1511,-1542,-1644, +1812	TATACA,ATTATA, TATAA,ATATAA, ATATAT,TATATAA,TATATA,TATAAAA,TATAAA,TAAAGATT,	core promoter element around -30 of transcription start
TCT-motif	-493	TCTTAC	part of a light responsive element
WUN-motif	+1162, +1395, +1227,+1548	AAATTTCTT	wound-responsive element

### Subcellular localization of BnMYB2 protein

**Fig 2:**
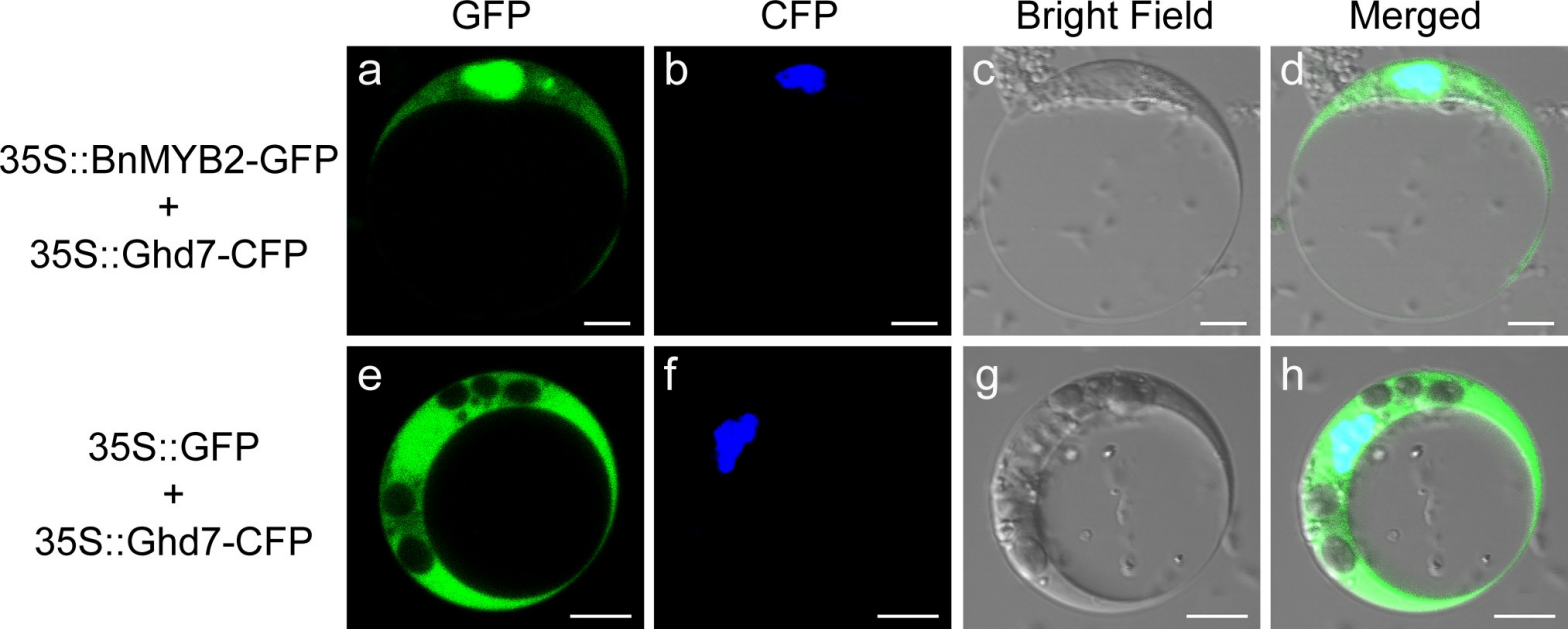
Subcellular localization of BnMYB2. Constructs 35S::BnMYB2-GFP and empty vector 35S::GFP were separately co-transformed into *Arabidopsis* protoplasts with nuclear marker 35S::OsGhd7-CFP. GFP and CFP fluorescences were observed using a laser confocal microscope. **a**, 35S::BnMYB2-GFP; **b**, 35S::OsGhd7-CFP; **c**, Bright field; **d**, Overlap images of (a), (b) and (c); **e**, 35S::GFP; **f**, 35S::OsGhd7-CFP; **g**, Bright field; **h**, Overlap images of (e), (f) and (g). The bar indicates 5 μm.

Using WoLF PSORT, BnMYB2 was predicted to be subcellularly localized to the nucleus. In order to verify the prediction, a transient expression vector pAN-BnMYB2 (BnMYB2 protein fused with GFP) was constructed and transformed into isolated *Arabidopsis* protoplasts. Microscopic observation showed that the green fluorescent signal of BnMYB2-GFP fusion protein was detected in the nucleus along with the cyan fluorescent signal of nuclear marker OsGhd7-CFP. In addition, a weak signal for BnMYB2-GFP was also observed in the cytoplasm. In contrast, green fluorescence was observed in the nucleus, cytoplasm and cytomembrane of cells expressing the GFP gene alone (Fig 2). The prediction and experimental observations indicated that the nucleus and cytoplasm are the main locations for BnMYB2 protein.

### *BnMYB2* expression patterns under Cd stress

**Fig 3:**
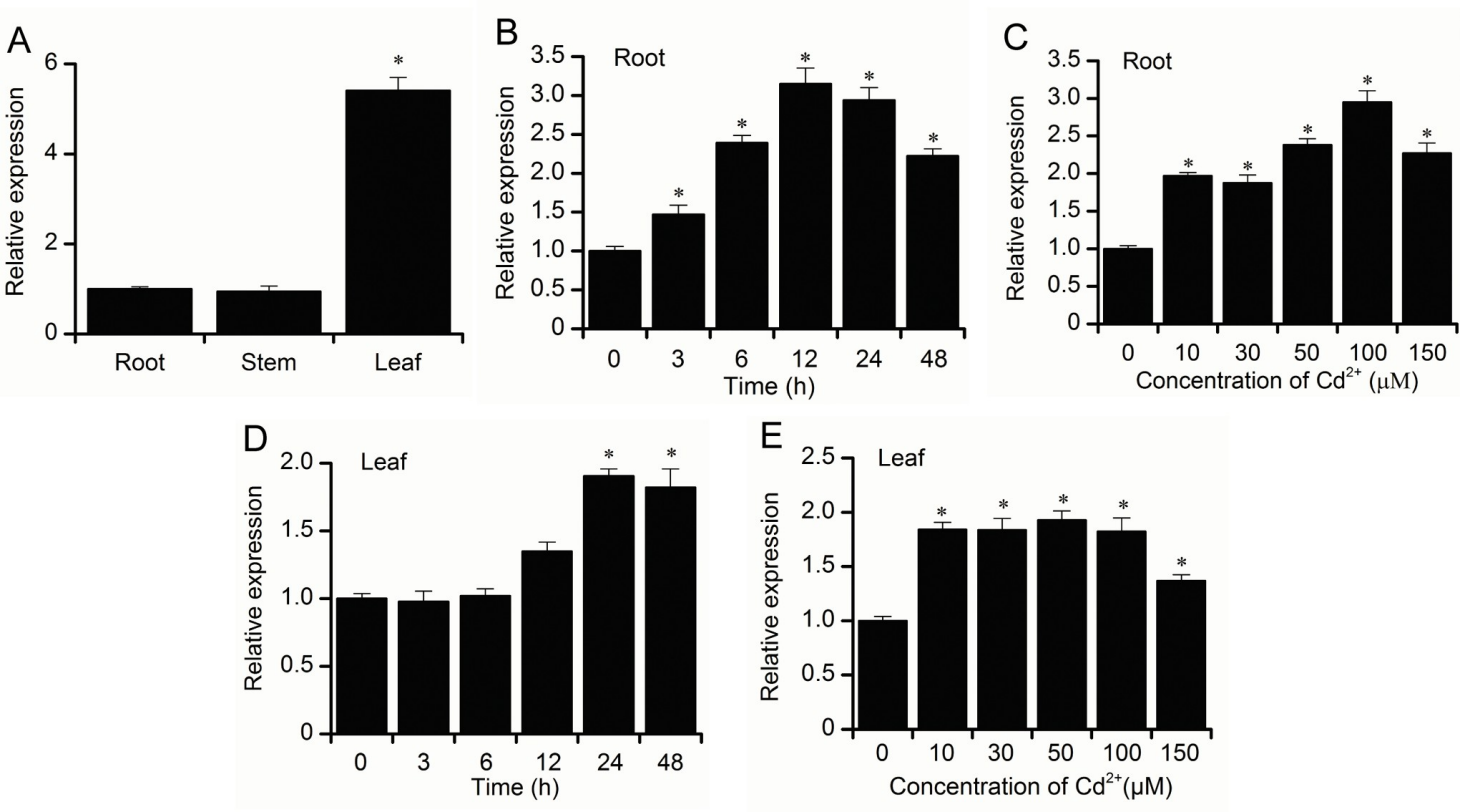
Expression pattern of *BnMYB2*. **A**, qRT-PCR analysis of *BnMYB2* transcript levels in roots, stems and leaves of 20-day-old ramie seedlings. **B**, *BnMYB2* transcript levels in the roots of ramie seedlings treated with 100 μM Cd for 0, 3, 6, 12, 24 and 48 h. **C,** *BnMYB2* transcript levels in the roots of ramie seedlings treated with 0, 10, 30, 50, 100 and 150 μM Cd for 24 h. **D**, *BnMYB2* transcript levels in the leaves of ramie seedlings treated with 100 μM Cd for 0, 3, 6, 12, 24 and 48 h. **E,** *BnMYB2* transcript levels in the leaves of ramie seedlings treated with 0, 10, 30, 50, 100 and 150 μM Cd for 24 h. Data are presented as the means of three biological replicates with SE shown by vertical bars. Asterisk indicates significantly difference (p<0.05) from the untreated group.

Real-time PCR experiments were carried out to study *BnMYB2* expression patterns in different *Boehmeria nivea* tissues and under Cd stress. The tissue specificity analysis showed that *BnMYB2* expression can be detected in the root, stem and leaf of ramie. *BnMYB2* expression was significantly higher in leaves than in roots and stems, and the expression level in roots and stems were similar (Fig 3A). Different *BnMYB2* expression patterns were detected when ramie seedlings were treated with Cd. The results indicated that *BnMYB2* expression in the roots and leaves were up-regulated significantly compared to the control (0 h or 0 μM Cd) after treatment with Cd. In time-course experiment, the greatest changes in expression were observed at 12 h in roots and 24 h in leaves, which resulted in a 3.2-fold increase (Fig 3B) and a 1.9-fold increase (Fig 3D), respectively. In dose-response experiment, the greatest changes in expression were observed at 100 μM Cd treatment in roots and 50 μM Cd treatment in leaves, which resulted in a 2.9-fold increase (Fig 3C) and a 1.9-fold increase (Fig 3E), respectively.

### Overexpression of *BnMYB2* improved the tolerance and accumulation of Cd in transgenic plants

In order to identify the function of *BnMYB2* in regulating plant response to Cd stress, the open reading frame of *BnMYB2* was inserted into a pBI121 vector with a 35S promoter to construct pBI121-BnMYB2, which was then transformed into Col-0 ecotype *Arabidopsis*. The transgenic *Arabidopsis* seedlings were screened using MS medium containing 30 mg·L^-1^ kanamycin (S3 Fig). To confirm expression of the exogenous *BnMYB2* in *Arabidopsis* plants, genomic DNA and total RNA were extracted from *Arabidopsis* plants (T1 generation) and analyzed by PCR and RT-PCR (S4 and S5 Figs). PCR results confirmed that the *BnMYB2* gene was integrated into the genome of transformed plants. RT-PCR results indicated that *BnMYB2* expression was highest in 35S::BnMYB2 carrying transgenic lines L3 and L6, which were selected for additional analysis.

**Fig 4:**
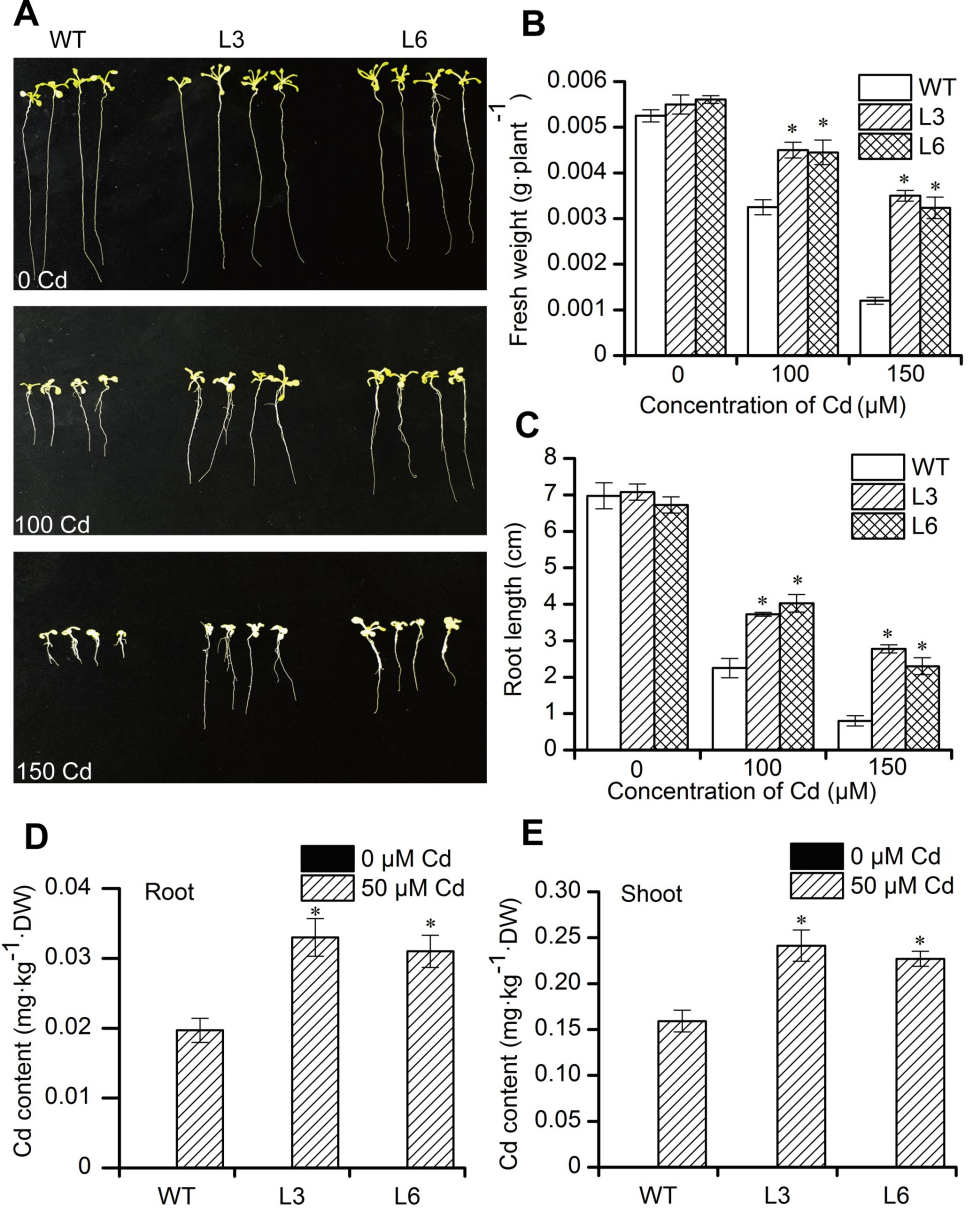
Analysis of the phenotype and Cd content of wild-type (WT) and *BnMYB2* transgenic *Arabidopsis* plants (L3 and L6) treated with Cd. **A**, Phenotype of *Arabidopsis* seedlings grown on MS medium with 0, 100 or 150 μM Cd for 14 d. **B**, Fresh weight of *BnMYB2* transgenic *Arabidopsis* seedlings grown on MS medium with 0, 100 or 150 μM Cd for 14 d. **C**, Root length of *BnMYB2* transgenic *Arabidopsis* seedlings grown on MS medium with 0, 100 or 150 μM Cd for 14 d. **D** and **E**, Concentration of Cd in roots and shoots of WT and transgenic *Arabidopsis* plants grown on soil irrigated with 0 or 50 μM Cd for 7 d. Data are presented as the means of three biological replicates with SE shown by vertical bars. Asterisk indicates significantly difference (p<0.05) from wild-type (WT).

Then, T3 seeds of WT (wild-type), L3 and L6 *Arabidopsis* were germinated and grown on MS solid medium containing Cd (0, 100 or 150 μM) for 14 days. In normal MS medium, no significant morphological differences were observed between WT, L3 and L6 lines (Fig 4A). When the seedlings were exposed to 100 or 150 μM Cd, the growth of WT, L3 and L6 lines were all significantly inhibited. However, the L3 and L6 transgenic lines both showed significantly better growth than WT. Under Cd exposure, the fresh weight of L3 and L6 transgenic plants root were 1.4–2.9 fold heavier than that of WT (Fig 4B). Similarly, the primary root elongation of L3 and L6 transgenic seedlings were 1.7–3.5 fold longer than that of WT when seedlings were exposed to Cd (Fig 4C). To examine the effect of *BnMYB2* gene expression on Cd accumulation in *Arabidopsis*, 30-day-old WT and transgenic *Arabidopsis* grown in soil were irrigated with half-strength Hoagland nutrient solution containing 0 μM or 50 μM Cd and allowed to grow for 7 days. Then the Cd content in roots and shoots were measured. In the presence of 0 μM Cd, the content of Cd in WT and transgenic *Arabidopsis* plants was undetectable because the soil and half-strength Hoagland nutrient solution were not contaminated by Cd. In the presence of 50 μM Cd, the two transgenic lines overexpressing *BnMYB2* showed significantly higher levels of Cd than WT plants in both roots and shoots. Cd content in the roots and shoots of transgenic lines was 1.6–1.7 and 1.4–1.5 fold higher than that of WT, respectively (Fig 4D and 4E). These results indicated that *BnMYB2* was able to enhance both Cd tolerance and accumulation in *Arabidopsis*.

## Discussion

As one of the largest plant transcription factor families, MYB transcription factors play important roles in the transcriptional regulation of plant growth, development and response to environment stress by employing their conserved DNA-binding domain to specifically bind the promoters of target genes [24, 46]. In the present work, we isolated a MYB transcription factor gene (named *BnMYB2*) in ramie using RACE and RT-PCR methods. The conserved domain analysis indicated that BnMYB2 protein contains one conserved MYB DNA-binding domain. Sequence alignment and phylogenetic tree analysis revealed that BnMYB2 shares high homology with known stress resistance-related 1R-MYB proteins from other plant species, such as TUS38128 [47], AtMYBS3 [48], OsMYBS3 [49], StMYB1R-1 [50] and GmMYB176 [51]. Among these proteins, AtMYBS3, a homolog of rice OsMYBS3, was reported to be activated by ABA and CdCl_2_. Thus, *BnMYB2* from ramie was predicted belonging to the 1R-MYB subfamily, and may play an important role in ramie adaption to adversity stress including Cd stress.

Furthermore, we obtained a 1947 bp promoter sequence of *BnMYB2* by PCR and analyzed this promoter sequence as detailed in additional file: S2 Fig. Sequence analysis showed that the promoter contains several stress-related hormone response elements, such as ABRE, CGTCA-motif, and P-box elements. Usually, plant tolerance to environmental stresses can be enhanced through the regulation of hormone metabolism and signal transduction to change morphology and physiological activities. For example, abscisic acid (ABA) plays an important role in plant response to diverse environmental abiotic stresses. Seo et al. [52, 53] reported that *AtMYB60* and *AtMYB96* act through the ABA signaling cascade to regulate drought stress and disease resistance. Abe et al. [54] confirmed that ABA could induce the expression of MYB gene *AtMYB2* in *Arabidopsis*, which was also induced by dehydration or salt stress. In plants, MeJA was shown to act as a signaling molecule in response to biotic and abiotic stresses [55]. Anjum et al. [56] found that the exogenous MeJA can improve soybean drought resistance by inducing alterations in lipid peroxidation and the antioxidative defense system. ABRE and CGTCA-motif elements found in the promoter indicated that *BnMYB2* expression may be induced by ABA and MeJA and involved in abiotic stress tolerance.

Initiating signaling cascades that regulate the expression of defense genes is an important way to manage plant's response to Cd [57]. Previous studies have shown that the expression of transcription factors belonging to MYB families is highly responsive to Cd stress [58]. About 20 percent of all MYB family genes in *Arabidopsis thaliana* are differentially expressed in response to Cd stress [33]. She et al. [40] studied the expression profile of Cd response genes in ramie roots using high throughput sequencing technology, finding that the expression of some MYB genes was significantly induced by Cd. Similarly, Liu et al. [59] identified ramie genes involved in Cd stress response using Illumina pair-end sequencing on two Cd-stressed plants grown on soil contaminated with 100 mg·kg^-1^ Cd, finding that a MYB3 transcription factor was up-regulated to 2.29-fold. The transcript level of *AIM1*, a MYB transcription factor in tomato, was induced about 1.5-fold under Cd stress (100 μM). In the present study, the transcript level of *BnMYB2* in ramie roots and leaves were significantly increased under Cd stress; the change in the expression of *BnMYB2* in roots was more obvious than that in leaves. The result indicated that *BnMYB2* has an important role in mediating Cd stress response in ramie, especially in roots.

The analysis of the subcellular localization of a protein can provide insight to its function. In the present study, BnMYB2 fused to GFP showed a strong signal in the nucleus and a weak signal in the cytoplasm when expressed in *Arabidopsis* protoplasts. Similar results also have been observed in other plant species. In *Salvia miltiorrhiza*, a R2R3 MYB transcription factor gene *SmMYB87* was found localized in the nucleus and cytomembrane of onion epidermal cells [60]. Several R3 MYB transcription factors such as ETC3, TCL1 and CPC related to trichome development in *Arabidopsis thaliana*, were found localized in the nucleus as well as in the cytoplasm when expressed in *Arabidopsis* protoplasts [61–63]. Isoflavonoids are natural compounds in plant which play important roles in plant growth, development and abiotic stress response. A 1R-MYB transcription factor GmMYB176 which regulates the biosynthesis of isoflavonoids in soybean, was found localized mainly in the nucleus and partially in the cytoplasm when expressed in tobacco leaf epidermal cell [64]. Meanwhile, GmMYB176 shares the nearest phylogenetic relationship with BnMYB2 (Fig 1B). In our study, BnMYB2 was localized both in nucleus and cytoplasm. The location of BnMYB2 in the nucleus implied that BnMYB2 is compatible with a role in transcriptional regulation of (as yet unknown) downstream target genes. However, the location of BnMYB2 in the cytoplasm is not yet understood. It could be due to an artifactual mislocalization due to the use of a 35S promoter to drive the expression of BnMYB2-GFP.

The functions of MYB proteins have been investigated in numerous plant species, such as *Arabidopis*, *Oryza sativa*, *Malus domestica* and maize, using genetic and molecular analysis. To illustrate that *BnMYB2* was able to regulate plant response to Cd stress, we investigated the effect of *BnMYB2* overexpression on Cd tolerance and accumulation in *Arabidopsis*. The *35S*::*BnMYB2* seedlings exhibited improved fresh weight, root elongation and higher Cd content compared to those of WT seedlings. These results above indicated that *BnMYB2* can mediate the Cd response in *Arabidopsis*. Some evidence have implicated MYB transcription factors in the accumulation of heavy metals in plants. Overexpression of *MYB49*, a Cd-induced MYB transcription factor in *Arabidopsis*, significantly increased Cd accumulation by directly regulating *HIPP22*, *HIPP44*, *bHLH38* and *bHLH101*. Whereas, the accumulation of Cd in *Arabidopsis* was significantly reduced when *MYB49* was knocked out [34]. *OsARM1*, an arsenite-responsive MYB transcription factor from rice, was able to regulate the As tolerance and uptake in rice by regulating the expression levels of As-associated transporters genes like *OsABCC1* by binding to their promoters [32]. Overexpression of a *Raphanus sativus* transcription factor gene *RsMYB1*, strongly enhance the tolerance of petunia to multi-heavy metals stress (K_2_Cr_2_O_7_, MnSO_4_, ZnSO_4_ and CuSO_4_) by enhancing the expression level of antioxidant genes (*CAT* and *SOD*) and abiotic stress-tolerant genes (*PCs* and *GSH*) [65]. Our results suggest that *BnMYB2* from ramie plays an important function in mediating Cd tolerance and accumulation in plants and provide new clues to explain the mechanisms of Cd tolerance and accumulation in plants.

## Conclusion

In this study, we isolated the *BnMYB2* gene, which belongs to the 1R-MYB transcription factor subfamily, from ramie. The *BnMYB2* promoter contains several cis-acting elements involved in phytohormone signaling and a variety of stress responses. It has a close phylogenetic relationship with other 1R-MYB transcription factors that contribute to different environment stress response. Analysis indicated that *BnMYB2* is localized to the nucleus and cytoplasm. *BnMYB2* transcription was significantly up-regulated by Cd stress. Heterologous overexpression of *BnMYB2* in *Arabidopsis* enhanced Cd tolerance and accumulation in transgenic plants.

## Supporting information

S1 Fig Nucleotide and deduced amino acid sequence of *BnMYB2* from *Boehmeria nivea*. Nucleotides were numbered on the left. The deduced amino acid residues were showed under the corresponding codons. Asterisk indicates the stop codon.
(DOC)

S2 Fig The DNA sequence of the *Boehmeria nivea* BnMYB2 promoter. The length of *BnMYB2* gene promoter sequence was 1 947 bp. The A of the ATG initiation codon is defined as +1. The CAAT-box, TATA-box, ABRE, ARE, CGTCA-motif, ERE, P-box, STRE, WUN-motif and other important cis-regulatory elements are boxed and labeled.
(DOCX)

S3 Fig Kanamycin resistant screening of *BnMYB2* transgenic *Arabidopsis thaliana* seedlings. T0 seeds of transgenic *Arabidopsis thaliana* seedlings were germinated on MS medium supplemented with 30 mg⋅L^-1^ kanamycin for screening. Transgenic lines remained green, while the WT type turned yellow.
(DOCX)

S4 Fig Identification of transgenic *BnMYB2* transgenic *Arabidopsis thaliana* seedlings by MYB2-35SF/MYB2-SPR primer. M: Trans2K Plus II DNA Marker; P: positive plasmid control; CK: no template negative control; WT: wild type *Arabidopsis thaliana* seedling; L1-L12: *BnMYB2* transgenic seedlings. The pBI121-BnMYB2 vectors were transferred by *Agrobacterium tumefaciens*-mediated genetic transformation into *Arabidopsis thaliana*. All overexpressing 35S::BnMYB2 transgenic lines (T1 generation) were verified by PCR using MYB2-35SF and MYB2-SPR primer.
(DOCX)

S5 Fig The relative expression of *BnMYB2* gene in transgenic *Arabidopsis thaliana*. WT: wild type *Arabidopsis thaliana* seedling; L2-L12: *BnMYB2* transgenic seedlings. Total RNA were extracted from leaves of overexpressing 35S:BnMYB2 transgenic lines (T2 generation) for qRT-PCR. *BnMYB2* transcript levels were significantly high in several transgenic lines, the overexpression effect is excellent in L3 and L6 lines. Data are presented as the means of three biological replicates with SE shown by vertical bars.
(DOCX)

S1 Table List of primers used in the study. (DOCX)

## Abbreviations

AAS: Atomic absorption spectroscopy
ABA: Abscisic acid
ABRE: Abscisic acid response elements
ARE: Anaerobic regulatory element
As: Arsenic
CAT: Catalase
CBF: C-repeat binding factors
Cd: Cadmium
CFP: Cyan fluorescent protein
Cu: Copper
ERE: Ethylene response element
GFP: Green fluorescent protein
GSH: glutathione
MeJA: Methyl jasmonate
MS: murashige and skoog
MYB: V-myb avian myeloblastosis viral oncogene homolog
ORF: open reading frame
Pb: Lead
PCR: Polymerase chain reaction
PCS: phytochelatin synthase
RACE: Rapid amplification of cDNA end
ROS: Reactive oxygen species
RT-PCR: Reverse transcription polymerase chain reaction
SANT: Switching-defective protein 3 [Swi3], adaptor 2 [Ada2], nuclear receptor co-repressor [N-CoR], transcription factor [TFIIB]
SOD: Superoxide dismutase
TFs: Transcription factors
UAP: Universal Amplification Primer
WT: wild-type
