# Supplementary material for: A MYB transcription factor, BnMYB2, cloned from ramie (Boehmeria nivea) is involved in cadmium tolerance and accumulation
Source: PLoS One. 2020 May 18;15(5):e0233375. doi: 10.1371/journal.pone.0233375 (PMC7233596; doi:10.1371/journal.pone.0233375)
Supplement: S1 Fig — Nucleotides were numbered on the left. The deduced amino acid residues were showed under the corresponding codons. Asterisk indicates the stop codon. (DOC) [file pone.0233375.s001.doc]

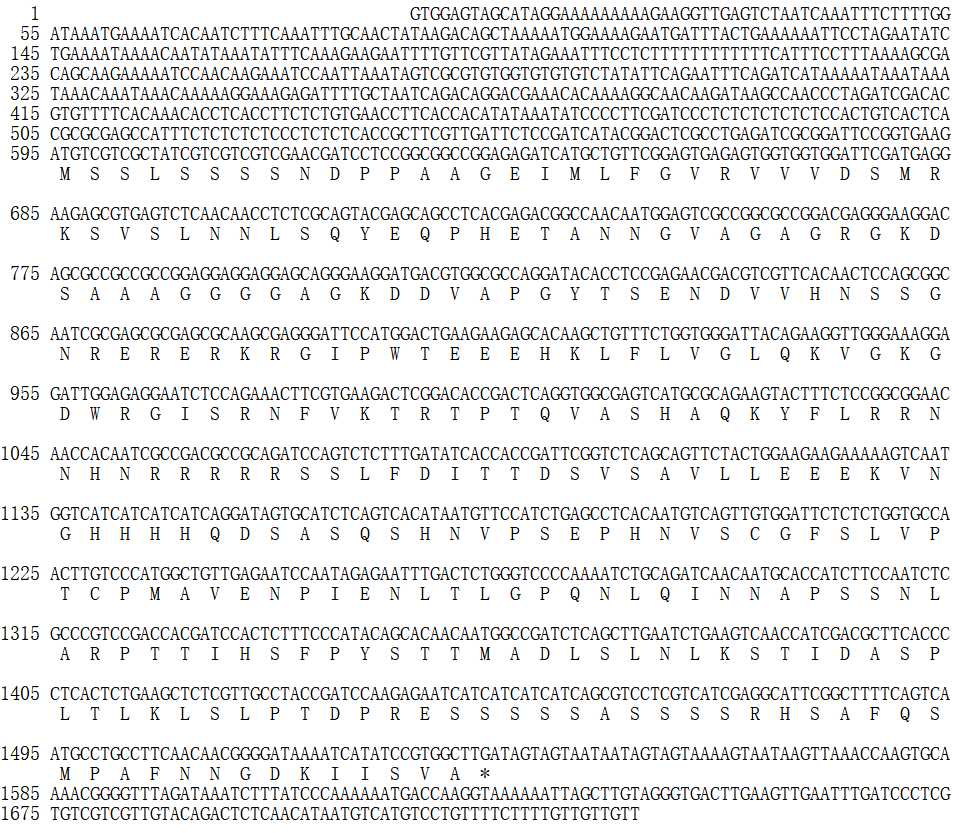


**S1 Fig. Nucleotide and deduced amino acid sequence of *BnMYB2* from *Boehmeria nivea.*** Nucleotides were numbered on the left. The deduced amino acid residues were showed under the corresponding codons. Asterisk indicates the stop codon.
