# Supplementary material for: A MYB transcription factor, BnMYB2, cloned from ramie (Boehmeria nivea) is involved in cadmium tolerance and accumulation
Source: PLoS One. 2020 May 18;15(5):e0233375. doi: 10.1371/journal.pone.0233375 (PMC7233596; doi:10.1371/journal.pone.0233375)
Supplement: S2 Fig — The length of BnMYB2 gene promoter sequence was 1 947 bp. The A of the ATG initiation codon is defined as +1. The CAAT-box, TATA-box, ABRE, ARE, CGTCA-motif, ERE, P-box, STRE, WUN-motif and other important cis-regulatory elements are boxed and labeled. (DOCX) [file pone.0233375.s002.docx]

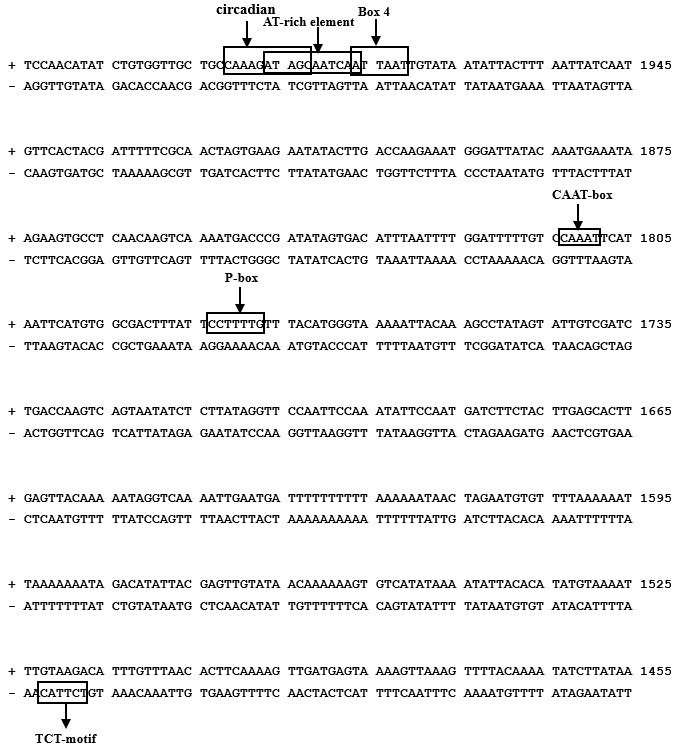

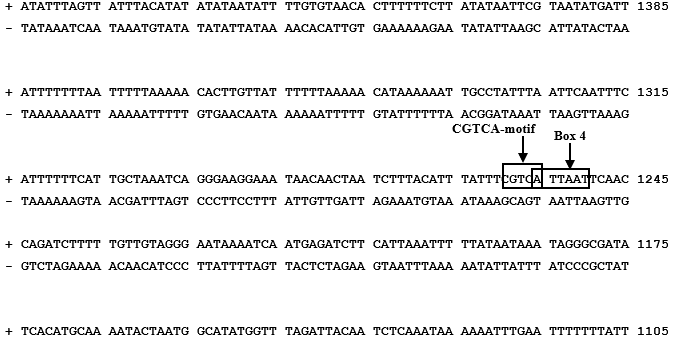

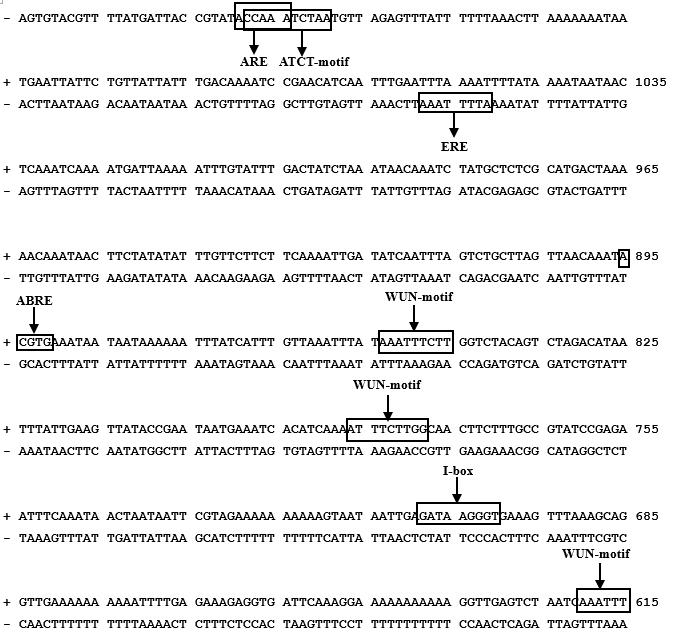

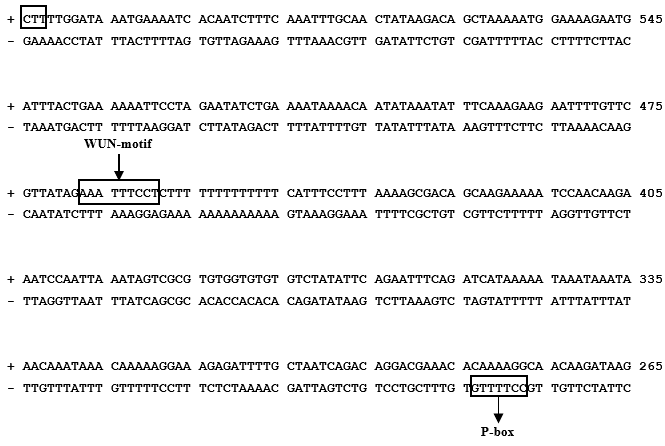

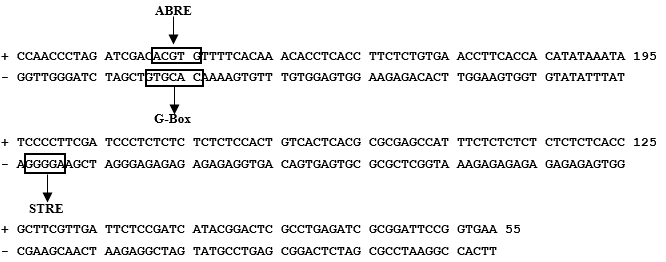


**S2 Fig. Sequence of *BnMYB2* promoter denoting the cis-elements predicted by PlantCARE database.** The length of *BnMYB2* gene promoter sequence was 1 947 bp. The A of the ATG initiation codon is defined as +1. The CAAT-box, TATA-box, ABRE, ARE, CGTCA-motif, ERE, P-box, STRE, WUN-motif and other important cis-regulatory elements are boxed and labeled.
