# Supplementary material for: A MYB transcription factor, BnMYB2, cloned from ramie (Boehmeria nivea) is involved in cadmium tolerance and accumulation
Source: PLoS One. 2020 May 18;15(5):e0233375. doi: 10.1371/journal.pone.0233375 (PMC7233596; doi:10.1371/journal.pone.0233375)
Supplement: S3 Fig — T0 seeds of transgenic Arabidopsis thaliana seedlings were germinated on MS medium supplemented with 30 mg⋅L-1 kanamycin for screening. Transgenic lines remained green, while the WT type turned yellow. (DOCX) [file pone.0233375.s003.docx]

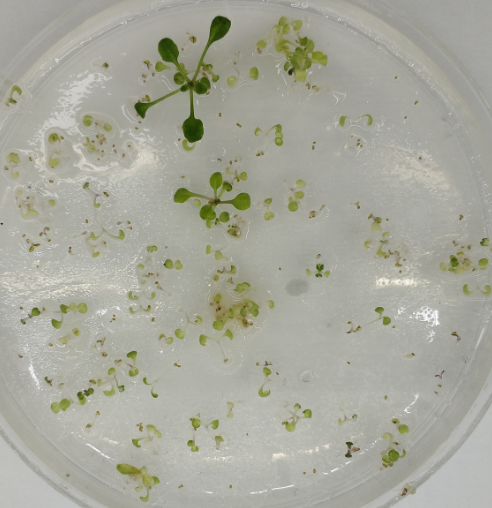

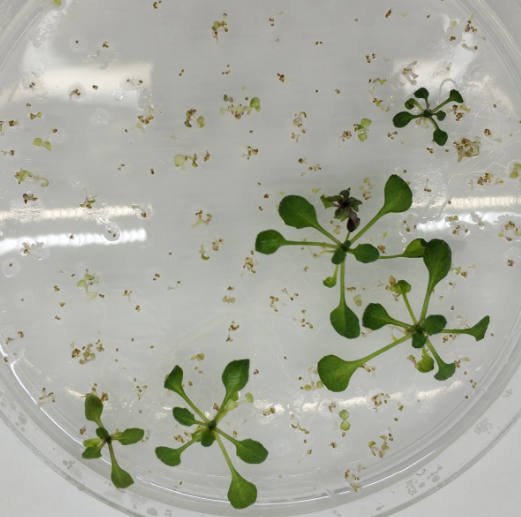


**S3 Fig. Kanamycin resistant screening of *BnMYB2* transgenic *Arabidopsis thaliana* seedlings.** T0 seeds of transgenic *Arabidopsis thaliana* seedlings were germinated on MS medium supplemented with 30 mg･L^-1^ kanamycin for screening. Transgenic lines remained green, while the WT type turned yellow.
