# Supplementary material for: A MYB transcription factor, BnMYB2, cloned from ramie (Boehmeria nivea) is involved in cadmium tolerance and accumulation
Source: PLoS One. 2020 May 18;15(5):e0233375. doi: 10.1371/journal.pone.0233375 (PMC7233596; doi:10.1371/journal.pone.0233375)
Supplement: S4 Fig — M: Trans2K Plus II DNA Marker; P: positive plasmid control; CK: no template negative control; WT: wild type Arabidopsis thaliana seedling; L1-L12: BnMYB2 transgenic seedlings. The pBI121-BnMYB2 vectors were transferred by Agrobacterium tumefaciens-mediated genetic transformation into Arabidopsis thaliana. All overexpressing 35S::BnMYB2 transgenic lines (T1 generation) were verified by PCR using MYB2-35SF and MYB2-SPR primer. (DOCX) [file pone.0233375.s004.docx]

L12

L7

L6

L4

L3

L2

L1

M

P

CK

WT


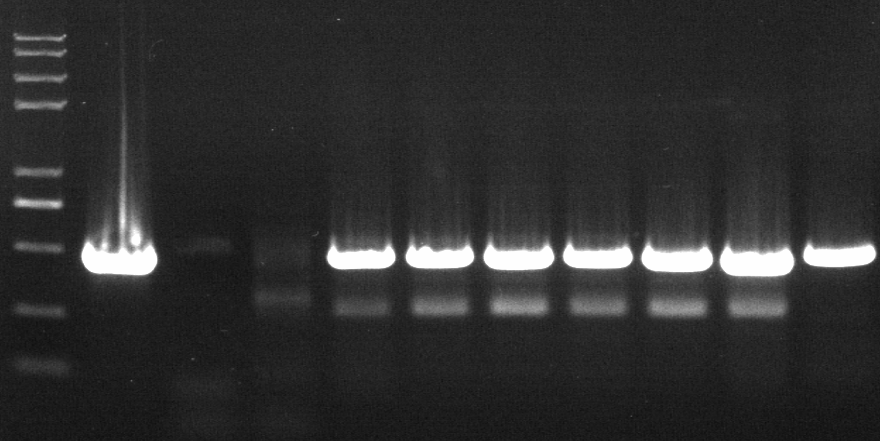


bp

750

500

250

**S4 Fig. Identification of transgenic *BnMYB2* transgenic *Arabidopsis thaliana* seedlings.** M: Trans2K Plus II DNA Marker; P: positive plasmid control; CK: no template negative control; WT: wild type *Arabidopsis thaliana* seedling; L1-L12: *BnMYB2* transgenic seedlings. The pBI121-BnMYB2 vectors were transferred by *Agrobacterium tumefaciens*-mediated genetic transformation into *Arabidopsis thaliana*. All overexpressing 35S::BnMYB2 transgenic lines (T1 generation) were verified by PCR using MYB2-35SF and MYB2-SPR primer.
