# Supplementary material for: A MYB transcription factor, BnMYB2, cloned from ramie (Boehmeria nivea) is involved in cadmium tolerance and accumulation
Source: PLoS One. 2020 May 18;15(5):e0233375. doi: 10.1371/journal.pone.0233375 (PMC7233596; doi:10.1371/journal.pone.0233375)
Supplement: S5 Fig — WT: wild type Arabidopsis thaliana seedling; L2-L12: BnMYB2 transgenic seedlings. Total RNA were extracted from leaves of overexpressing 35S:BnMYB2 transgenic lines (T2 generation) for qRT-PCR. BnMYB2 transcript levels were significantly high in several transgenic lines, the overexpression effect is excellent in L3 and L6 lines. Data are presented as the means of three biological replicates with SE shown by vertical bars. (DOCX) [file pone.0233375.s005.docx]

**S5 Fig. The relative expression of *BnMYB2* gene in transgenic *Arabidopsis thaliana*.** WT: wild type *Arabidopsis thaliana* seedling; L2-L12: *BnMYB2* transgenic seedlings. Total RNA were extracted from leaves of overexpressing 35S:BnMYB2 transgenic lines (T2 generation) for qRT-PCR. *BnMYB2* transcript levels were significantly high in several transgenic lines; the overexpression effect is excellent in L3 and L6 lines. Data are presented as the means of three biological replicates with SE shown by vertical bars.
