# Supplementary material for: A MYB transcription factor, BnMYB2, cloned from ramie (Boehmeria nivea) is involved in cadmium tolerance and accumulation
Source: PLoS One. 2020 May 18;15(5):e0233375. doi: 10.1371/journal.pone.0233375 (PMC7233596; doi:10.1371/journal.pone.0233375)
Supplement: S1 Table — (DOCX) [file pone.0233375.s006.docx]

**S1 Table. List of primers used in the study**

| No. | Primer name | Direction | Sequence (5′→3′) |
| --- | --- | --- | --- |
| 1 | MYB2-3FO | Forward | CTCACCGCTTCGTTGATTCTCC |
| 2 | MYB2-3FI | Forward | CGATCATACGGACTCGCCTGAG |
| 3 | MYB2-5FO | Reverse | GATTACGCCAAGCTTGGGAAAGAGTGGATCGTGGTCGG |
| 4 | MYB2-5FI | Reverse | GATTACGCCAAGCTTCCACCACCACTCTCACTCCGAACAG |
| 5 | MYB2-F | Forward | CTCTCACCGCTTCGTTGATT |
| 6 | MYB2-R | Reverse | CCCGTTTTGCACTTGGTTTAA |
| 7 | MYB2-PF | Forward | TTCCAACATATCTGTGGTTGCTGC |
| 8 | MYB2-PR | Reverse | CCGTCTCGTGAGGCTGCTC |
| 9 | MYB2-qF | Forward | TCACAACTCCAGCGGCAATC |
| 10 | MYB2-qR | Reverse | TCCCAACCTTCTGTAATCCCAC |
| 11 | BnActin-F | Forward | GTTGAACCCTAAGGCTAACAGAG |
| 12 | BnActin-R | Reverse | GGAATCCAGCACGATACCAG |
| 13 | MYB2-XbaI-F | Forward | AGTCCGGAGCTAGCTCTAGACCGGTGAAGATGTCGTCGC |
| 14 | MYB2-BamHI-R | Reverse | CCCTTGCTCACCATGGATCCAGCCACGGATATGATTTTATCCC |
| 15 | MYB2-BamHI-F | Forward | CGGGATCCATGTCGTCGCTATCGTCGTCGTCGAACGATCC |
| 16 | MYB2-SacI-R | Reverse | CGAGCTCTCAAGCCACGGATATGATTTTATCCCCGTTGTTGAAGGC |
| 17 | MYB2-35SF | Forward | AAGGCCATCGTTGAAGATGCCTCTG |
| 18 | MYB2-SPR | Reverse | CGCGATTGCCGCTGGAGTTG |
